# Meal rich in rapeseed oil increases 24-h fat oxidation more than meal rich in palm oil

**DOI:** 10.1371/journal.pone.0198858

**Published:** 2018-06-14

**Authors:** Katsuhiko Yajima, Kaito Iwayama, Hitomi Ogata, Insung Park, Kumpei Tokuyama

**Affiliations:** 1 Doctoral Program in Sports Medicine, Graduate School of Comprehensive Human Sciences, University of Tsukuba, Tsukuba, Ibaraki, Japan; 2 Department of Administrative Nutrition, Faculty of Health and Nutrition, Tokyo Seiei College, 4–6 Nishishinkoiwa 1-chome, Katsushika-ku, Tokyo, Japan; 3 Hiroshima University, Graduate School of Integrated Arts and Sciences, Higashi-Hiroshima, Japan; 4 International Institute for Integrative Sleep Medicine, University of Tsukuba, Tsukuba, Ibaraki, Japan; University of Illinois, UNITED STATES

## Abstract

The fatty acid composition of the diet has been linked to the prevalence of diabetes and cardiovascular diseases. Compared with monounsaturated fatty acids, saturated fatty acids decrease fat oxidation and diet-induced thermogenesis. A potential limitation of previous studies was the short duration (≦5h) of calorimetry used. The present study compared the effects of a meal rich in saturated and unsaturated fatty acids on 24-h of fat oxidation. Ten males participated in two sessions of indirect calorimetry in a whole-room metabolic chamber. At each session, subjects consumed three meals rich in palm oil (44.3% as saturated, 42.3% as monounsaturated and 13.4% as polyunsaturated fatty acid) or rapeseed oil (11.7% as saturated, 59.3% as monounsaturated and 29.0% as polyunsaturated fatty acid). Fat oxidation over 24-h was significantly higher in the meal rich in rapeseed oil (779 ± 202 kcal/day) than that rich in palm oil (703 ± 158 kcal/day, P < 0.05), although energy expenditure was similar between both meal conditions. Meal rich in unsaturated fatty acids increased the oxidation of exogenous and/or endogenous fat. The results of a long calorimetry period indicate that rapeseed oil offered an advantage toward increased 24-h fat oxidation in healthy young males.

## Introduction

The amounts of fat intake and number of patients who have developed lifestyle-related diseases have increased in parallel in developed countries [1–3]. In addition to quantity of fat intake, quality of fat has been identified as a culprit for the increased prevalence of diabetes and cardiovascular diseases, as outlined here [4–7]. Epidemiological studies identified a link between adherence to the Mediterranean diet and health outcomes such as reduced mortality, cardiovascular diseases and some cancers [8–10]. In terms of fatty acid composition, the intake of dietary saturated fatty acids seems to exacerbate these diseases, while the monounsaturated fatty acid intake seems to ameliorate them [4–7]. Weight gain is also related to dietary fatty acid composition. Association of weight gain with percentage of calories from saturated fat is stronger than that with total fat intake [11–14]. During a 7-week dietary intervention study, meals rich in polyunsaturated and monounsaturated fatty acid decreased liver fat and total body fat compared to those rich in saturated fatty acids [15]. When meals rich in monounsaturated fatty acids were consumed for 18 months in a clinical intervention trial, body weight of obese subjects was markedly decreased (−4.1 kg), while other subjects who consumed a low fat diet increased their body weight (2.9 kg) [16]. Despite the same caloric intake being provided to subjects, differences in weight gain during the intervention study [16] suggest that energy expenditure is also affected by dietary fatty acid composition.

Studies using indirect calorimetry show that the effects of dietary fatty acid composition on energy expenditure after consumption of a single meal are ambiguous. One study reported an increased energy expenditure after a meal rich in unsaturated fatty acids [17] while other studies failed to observe a difference in energy expenditure between meals rich in saturated and unsaturated fatty acids [18–20]. On the other hand, fat oxidation was higher after a meal rich in unsaturated fatty acids compared to that after a meal rich in saturated fatty acids in these studies [17–20]. Of note, the duration of indirect calorimetry (5 h) may not have been long enough to account for the entire acute effect of a meal on energy metabolism [17–20]. Studies using fatty acids labelled with stable isotopes showed that oxidation of exogenous fatty acids continue for at least 9 h [21]. Different forms of fat (olive oil, dairy products or walnuts) may affect the time course of fat digestion and absorption [22, 23], and it becomes critical when the observation period after fat consumption is limited. Furthermore, differences in appearance and taste of experimental meals prevent the use of a blind study protocol.

The aim of the present study was to compare the effects of meals rich in saturated and unsaturated fatty acids on 24-h fat oxidation. Muffins made with either palm or rapeseed oil were used as staple foods with different fatty acid compositions as part of the experimental meal, enabling us to design a single-blind experimental protocol.

## Experimental methods

### Subjects

Ten healthy young males were recruited in this study after providing written, informed consent. Body composition was measured using the bioimpedance method (BC-118E, TANITA Co., Tokyo, Japan). On average, subjects were 25.3 ± 1.0 years of age, 171.7 ± 1.6 cm in height, 67.2 ± 3.6 kg in body weight and had 16.3% ± 1.7% body fat. All subjects were non-smokers and free of pathological conditions, and none were taking medications or supplements. This study was conducted according to the guidelines laid out by the Declaration of Helsinki and all procedures involving human subjects were approved by the Ethics Committee of the University of Tsukuba (approval number 27–71). All subjects provided written informed consent before the study commencement; the protocol was registered with Clinical Trials UMIN, ID no.: 00028960.

### Protocol

The study was a randomised, single-blind, cross-over design including 24-h of indirect calorimetry with three meals rich in palm or rapeseed oil. A washout period of 3–10 days was included between trials.

Subjects were instructed not to change habitual lifestyle (i.e. bedtime and wake-up time) and to refrain from excess alcohol consumption between trials. On the day before measurement, subjects were asked to abstain from exercise and consumption of alcohol and caffeine. Subjects ingested their normal meals three times a day and meal-time was confirmed by a self-reported diary. Subjects entered the metabolic chamber the day prior to the 24-h indirect calorimetry session (day 1, 22:00). Once in the metabolic chamber, subjects slept for 8 h, from 23:00 to 7:00. On day 2, three experimental meals (breakfast at 8:00, lunch at 12:30 and dinner at 19:00) were provided. Energy metabolism was measured until the next morning, at 7:00 (day 3).

### Normal and experimental meals

On day 1, normal meals were individually standardised based on the estimated energy requirement for Japanese adults [24], assuming a physical activity factor of 1.75. The staple food of the normal meals was rice and the average energy content of the normal meals was 2625 ± 132 kcal/day. Expressed as a percentage of total energy, meals contained 15% protein, 24% fat and 61% carbohydrate.

On day 2, experimental meals were prepared assuming a physical activity factor of 1.3. The staple food of the experimental meals was a muffin made with palm oil (high-saturated fatty acid meal) or rapeseed oil (high-unsaturated fatty acid meal). Palm or rapeseed oil (8 g) was mixed with wheat flour (20 g), egg (11 g), sugar (3 g) and water (8 g), and baked in oven at 180°C for 25 min. The subjects were provided with side dishes in addition to muffins as staple food. Expressed as a percentage of total energy, experimental meals had a macronutrient profile of 15% protein, 42% fat and 43% carbohydrate. Energy content of the experimental meals was 2085 ± 101 kcal/day, and the fatty acid composition of the experimental meals is shown in Table 1.

**Table 1:** Composition of experimental meals.

	Meal rich in palm oil	Meal rich in rapeseed oil
Caloric distribution (% of energy)		
Protein	14.5	14.5
Fat	42.1	42.1
Carbohydrate	43.4	43.4
Fatty acid profile (g/100 g)		
Palmitic	37.6	7.8
Oleic	41.6	57.6
Linoleic	12.5	19.9
Stearic	4.9	2.9
α-Linolenic	0.7	8.7
Myristic	1.0	0.2
Palmitoleic	0.5	0.5
Eicosapentaenoic	0.0	0.0
Docosahexaenoic	0.1	0.1
Arachidonic	0.2	0.2
Fatty acid class (%)		
Saturated	44.3	11.7
Monounsaturated	42.3	59.3
Polyunsaturated	13.4	29.0

### Indirect calorimetry

Energy metabolism was measured using a room-sized metabolic chamber (Fuji Medical Science, Chiba, Japan). The airtight chamber measured 2.00 × 3.45 × 2.10 m, with an internal volume of 14.49 m^3^. The temperature and relative humidity of the incoming air were controlled at 25.0°C ± 0.5°C and 55.0% ± 3.0%, respectively. Concentrations of oxygen (O_2_) and carbon dioxide (CO_2_) in the outgoing air were measured using an online process mass spectrometer (VG Prima δB, Thermo Electron, Winsford, UK). Precision of the mass spectrometer, defined as the standard deviations for continuous measurement of calibration gas mixture (O_2_ 15%, CO_2_ 5%), were 0.0016% and 0.0011% for O_2_ and CO_2_, respectively. Every minute, O_2_ consumption (VO_2_) and CO_2_ production (VCO_2_) rates were calculated using an algorithm for improved transient response [25]. Metabolic chamber was calibrated by ethanol combustion test and intermittent gas infusion test [25].

Macronutrient oxidation and energy expenditure were calculated from VO_2_, VCO_2_ and urinary nitrogen excretion (N) [26]. Rates of N, an index of protein oxidation, were assumed to be constant during the calorimetry. Equations for glucose, fat and protein oxidation rates were as follows:

$$\mathrm{Glucose}\;\mathrm{oxidation}\;\left(\frac{g}{\min}\right)=4.55\;\dot{V}CO_{2}\left(\frac{L}{\min}\right)-3.21\;\dot{V}O_{2}\left(\frac{L}{\min}\right)-2.87\;\dot{N}\;\left(\frac{g}{\min}\right)$$

$$\mathrm{Fat}\;\mathrm{oxidation}\;\left(\frac{g}{\min}\right)=1.67\;\dot{V}O_{2}\;\left(\frac{L}{\min}\right)-1.67\;\dot{V}{\mathrm{CO}}_{2}\;\left(\frac{L}{\min}\right)-1.92\;\dot{N}\;\left(\frac{g}{\min}\right)$$

$$\mathrm{Protein}\;\mathrm{oxidation}\;\left(\frac{g}{\min}\right)=6.25\;\dot{N}\;\left(\frac{g}{\min}\right)$$

Once the rates of glucose, fat and protein oxidation were computed, the total rate of energy production could be estimated by taking into account the caloric equivalent of the three substrates. Conversion factors for energetic equivalents were 4.10 kcal/g for protein (25.625 kcal/g for urinary N), 3.74 kcal/g for carbohydrate and 9.50 kcal/g for fat [26].

### Physical activity

Physical activity was measured with a uniaxial accelerometer activity monitor (ActiGraph, Ambulatory Monitoring Inc., Ardsley, NY, USA). All subjects wore the wristwatch accelerometer in zero-crossing mode [27]. Gross motor activity of the accelerometer was estimated at 1-min intervals.

### Autonomic nervous system activity

R–R intervals of the electrocardiogram were continuously monitored using a telemetric heart rate monitor (LX-3230, Fukuda Denshi Co., Ltd., Tokyo, Japan) and the power spectrum of heart rate variability was estimated using the maximum entropy method. The spectral measured were computed as amplitudes (i.e. areas under the power spectrum) and were presented in square milliseconds (ms^2^). Parasympathetic and sympathetic nervous system activity were estimated as high frequency (HF; 0.15–0.4 Hz) and as a power ratio of low frequency (LF; 0.04–0.15 Hz) to high frequency (LF/HF), respectively [28].

### Statistical analysis

Results are expressed as the mean ± the standard deviation of the mean. A study group of ten subjects was required for a power of 80% at a two-sided alpha of 0.05. We performed a power analysis; the actual power was greater than 80% for each comparison. Paired *t*-test was used for comparison of total energy expenditure, carbohydrate oxidation and fat oxidation, and averages of respiration quotient, heart rate and autonomic nervous system activity during the 24-h period. To compare the time courses of energy metabolism and autonomic nervous system activity in the two trials, mean values after breakfast (8:00–12:00 hours), lunch (12:30–16:30 hours) and dinner (19:00–23:00 hours) were calculated for each subject, and repeated-measures two-way analysis of variance (ANOVA) was used. When ANOVA revealed a significant main effect (trial and time) or interaction (trial × time), the Bonferroni test was performed for a post hoc analysis to identify differences. Differences in postprandial fat oxidation between the two dietary conditions were compared among breakfast, lunch and dinner using repeated-measures one-way ANOVA. Statistical analyses were performed using SPSS statistical software (version 23.0; SPSS Japan, Tokyo, Japan), with the level of statistical significance set at 5%.

## Results

All subjects completed the two trials and there were no significant differences in subjects’ body mass, body fat, or fat-free mass between the trials. Energy balance was slightly positive in both dietary condition, but there were no statistical differences between the conditions (Table 2).

**Table 2:** Energy balance, energy metabolism and autonomic nervous system activity in male subjects during 24-h of calorimetry (n = 10)^a^.

Parameters	Meal rich in palm oil	Meal rich in rapeseed oil	P-value
Energy intake (kcal/24h)	2085 ± 318	2085 ± 318	
Energy expenditure (kcal/24h)	2046 ± 275	2059 ± 264	0.519
Energy balance (kcal/24h)^b^	39 ± 126	26 ± 135	0.519
Respiration quotient (/24h)	0.866 ± 0.013	0.859 ± 0.020	0.046*
Carbohydrate oxidation (kcal/24h)	1057 ± 172	1020 ± 175	0.107
Fat oxidation (kcal/24h)	703 ± 158	779 ± 202	0.047*
Protein oxidation (kcal/24h)	285 ± 43	261 ± 110	0.500
Heart rate (beats/min)	62 ± 7	60 ± 8	0.057
Parasympathetic nervous system activity (ms^2^/min)	935 ± 476	1040 ± 512	0.015*
Sympathetic nervous system activity LF/HF (/min)	2.7 ± 1.6	2.8 ± 1.7	0.329

^a^Statistical analyses was performed by paired *t*-test.

^b^Energy balance was calculated by subtracting energy expenditure from energy intake for each subject.

Abbreviations: LF/HF, low frequency to high frequency.

*P < 0.05.

Between the two trials, there were no statistically significant differences in 24-h energy expenditure, carbohydrate oxidation or protein oxidation (Table 2). Accumulated fat oxidation during the 24-h period was higher and the average respiration quotient during the 24-h calorimetry period was lower in the meal rich in rapeseed than the meal rich in palm oil (Table 2).

**Fig 1:**
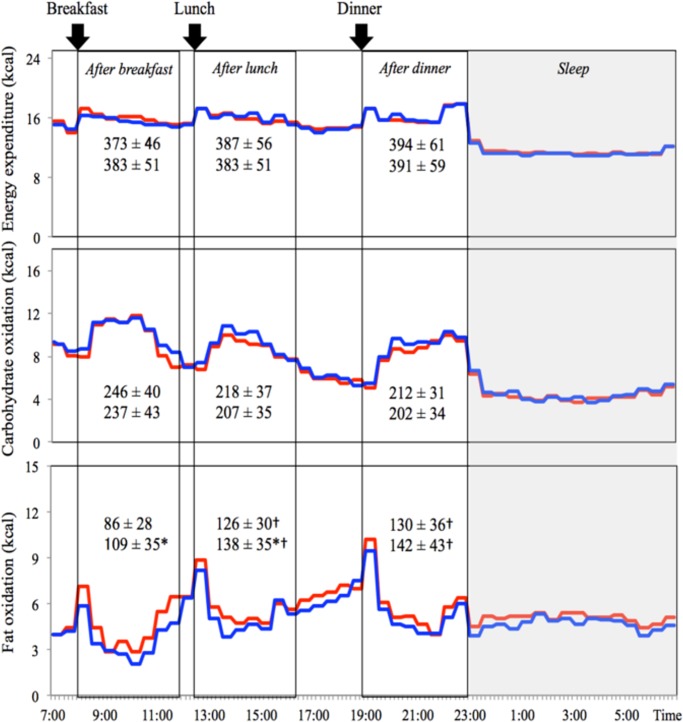
Diurnal variations of energy metabolism. Mean values of 10 subjects were plotted at 30-min intervals for the meals rich in rapeseed oil shown with red line and palm oil shown with blue line. Mean values after breakfast (8:00–12:00 hours), lunch (12:30–16:30 hours) and dinner (19:00–23:00 hours) are also shown. Values in the figure show accumulated energy expenditure and substrate oxidation over 4 h after breakfast, lunch and dinner. Statistical analyses were performed on data at each meal using repeated-measures two-way analysis of variance, followed by Bonferroni post hoc tests. *P < 0.05 vs. palm oil. †P < 0.05 vs. breakfast within trial.

Fig 1 shows diurnal variation of energy metabolism during 24-h of two trials and values in the figure show accumulated energy expenditure and substrate oxidation over 4 h after breakfast, lunch and dinner. Significant main effects of trial and time on the 4 h post-prandial period were observed only in fat oxidation (P < 0.05), but there were no significant main effects of trial and time on post-prandial energy expenditure and carbohydrate oxidation. When the meal rich in rapeseed oil was consumed, fat oxidation was significantly higher after breakfast and lunch than when the meal based on palm oil was consumed. However, there were no significant differences in fat oxidation after dinner between the two trials (Fig 1).

**Fig 2:**
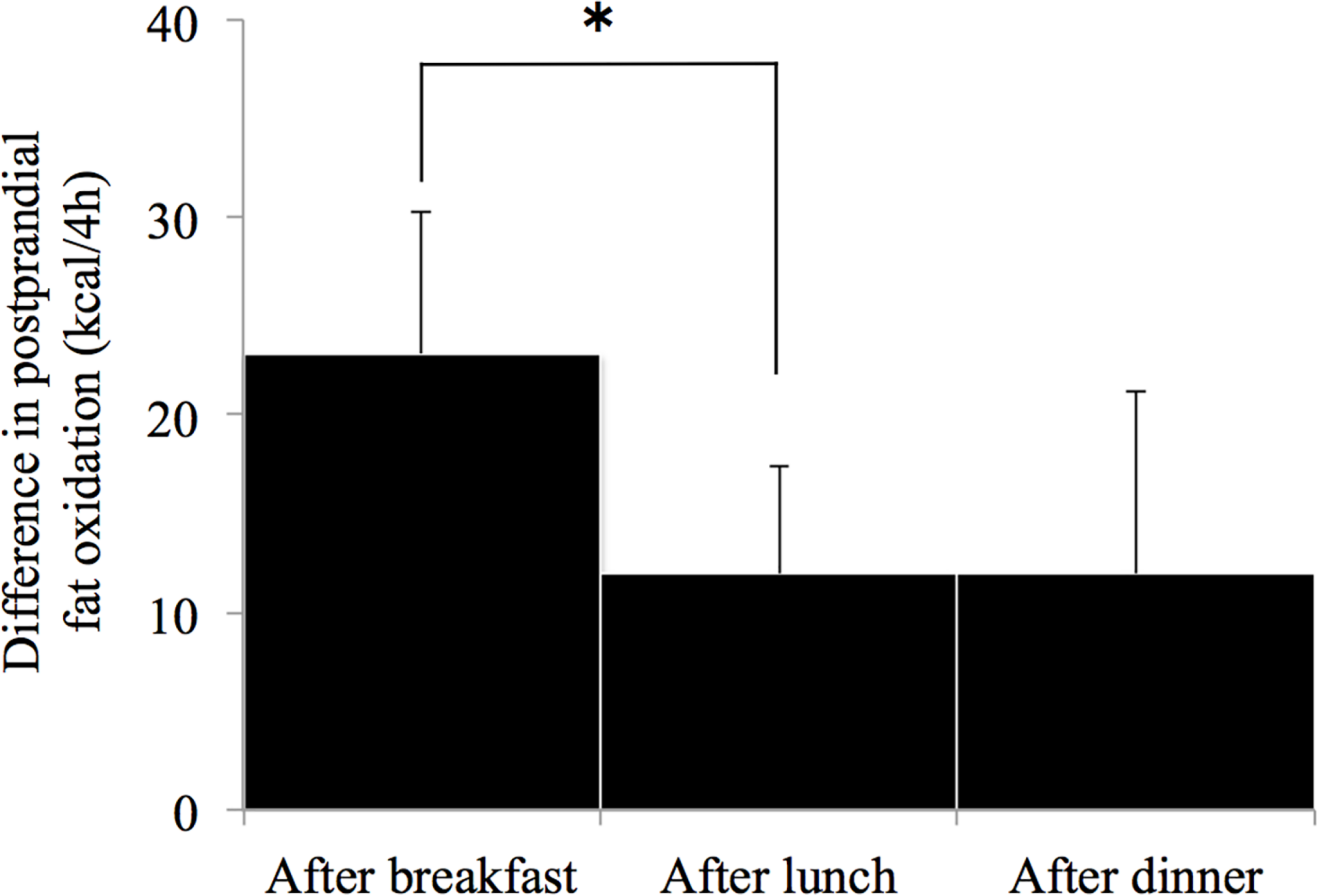
Difference in postprandial fat oxidation between the two conditions at each meal. The difference in postprandial fat oxidation at each meal was calculated by subtracting fat oxidation over 4 h after the meal rich in palm oil from that of the meal rich in rapeseed oil. Mean values of the difference in postprandial fat oxidation after breakfast, lunch and dinner are shown in the bar graph. Statistical analyses were performed using repeated-measures one-way analysis of variance, followed by Bonferroni post hoc tests. *P < 0.05 vs. breakfast.

The differences in postprandial (4 h) fat oxidation between the two dietary conditions were 23 ± 7 kcal/4 h after breakfast, 12 ± 6 kcal/4 h after lunch and 12 ± 9 kcal/4 h after dinner (Fig 2). The difference in postprandial fat oxidation after breakfast was significantly larger than that after lunch.

**Fig 3:**
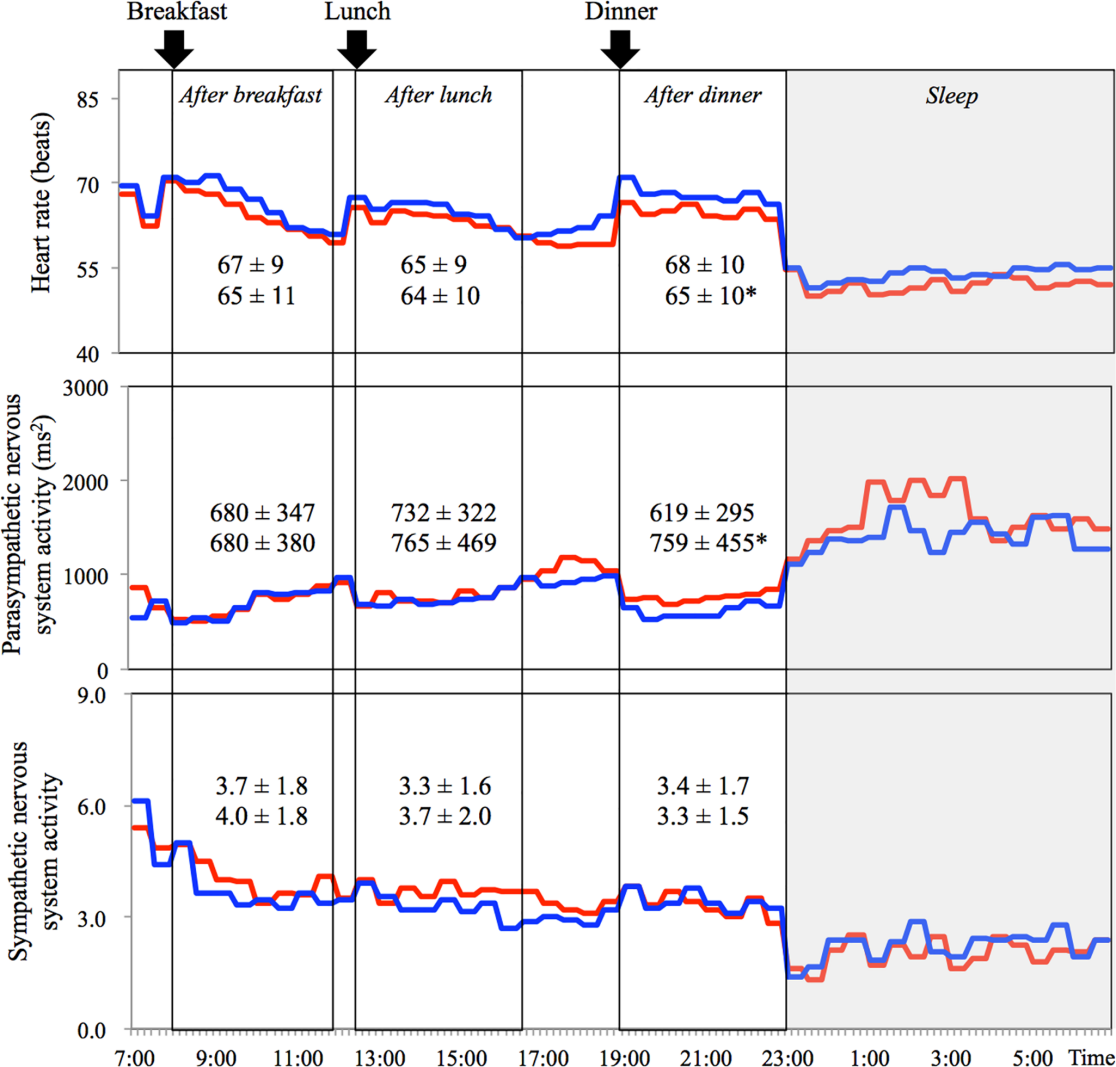
Diurnal variations of heart rate and autonomic nervous system activity. Mean values of 10 subjects were plotted at 30-min intervals for the meal rich in rapeseed oil shown with red line and palm oil shown with blue line. Mean values after breakfast (8:00–12:00 hours), lunch (12:30–16:30 hours) and dinner (19:00–23:00 hours) are also shown. Statistical analyses were performed on averages over 4 h after each meal by repeated-measures two-way ANOVA, followed by Bonferroni post hoc tests. Columns connected by line are statistically different (*P < 0.05).

Average sympathetic nervous system activity over 24-h was similar between the two trials. Average parasympathetic nervous system activity was higher in the meal rich in rapeseed oil than that rich in palm oil (Table 2). As a result, average heart rate over 24-h tended to be lower in the meal rich in rapeseed oil than that rich in palm oil (Table 2). Two-way ANOVA revealed that heart rate was lower [main effect of trial, P < 0.05] and parasympathetic nervous system activity was higher [main effect of trial, P < 0.05] after dinner in the meal rich in rapeseed oil than that rich in palm oil (Fig 3).

Physical activity was not significantly different between the two dietary conditions during day 1 (100.6 ± 14.8 counts/min for rapeseed oil vs. 99.4 ± 8.0 counts/min for palm oil, ns) and day 2 (69.2 ± 18.8 counts/min for rapeseed oil vs. 65.1 ± 4.3 counts/min for palm oil, ns).

## Discussion

The main finding of the present study was that, compared with the meal rich in palm oil, the meal rich in rapeseed oil increased 24-h fat oxidation without affecting energy expenditure. The present results are consistent with previous findings derived from 5 h of indirect calorimetry [17–20]. The time course of fat oxidation during 24-h of indirect calorimetry in the present study revealed new insight into the effect of dietary intervention on whole body fat oxidation. First, the promotion of fat oxidation by the meal rich in rapeseed oil was detected after breakfast and lunch (Fig 1). In addition, the enhancement of fat oxidation after breakfast was greater than that after lunch and dinner (Fig 2). Differences in postprandial fat oxidation between the two dietary conditions were statistically significant after breakfast and lunch. The following explanation may account for the lack of effect of the experimental meal after dinner. First, since subjects consumed a high-fat meal at breakfast, lunch and dinner, fat oxidation was gradually elevated during the daytime. As a result of the ceiling effect, differences in fat oxidation between the two conditions after dinner may not have been observed. Second, if the oxidation rate of monounsaturated fatty acid was simply faster than that of saturated fatty acid, the amount of fat oxidation in the saturated fatty acid (i.e. palm oil) condition would have caught up with that of the monounsaturated fatty acid (i.e. rapeseed oil) condition after dinner. However, this was unlikely to have been the case since the differences in accumulated fat oxidation remained significant at the end of 24-h of calorimetry.

Although the underlying mechanism by which the meal rich in rapeseed oil enhances fat oxidation remains unknown, several possibilities exist. First, estimates of fat oxidation by indirect calorimetry consist of oxidation of exogenous and endogenous fat (dietary fat and body fat, respectively). It is possible that rapeseed oil was an excellent substrate for oxidation. Consistent with this possibility, a study using isotope-labelled fatty acids has shown that the enrichment of ^13^CO_2_ from long-chain fatty acids over 9 h was higher with increasing degrees of unsaturation [21]. After digestion and assimilation, monounsaturated fatty acid is packaged in chylomicrons, the size of which is larger than that of saturated fatty acids [29]. Larger chylomicrons are hydrolysed by lipoprotein lipase better than are smaller chylomicrons [30]. Monounsaturated fatty acid is taken up by the peripheral tissues via fatty acid transporters and fatty acid-binding protein more quickly than is saturated fatty acid [30, 31]. Secondly, rapeseed oil acted as a stimulant of endogenous fat oxidation. Peroxisome proliferator-activated receptors (PPARs) provide a molecular mechanism for the modulation of fat utilisation and thermogenesis by different types of fatty acids [32]. Activation of PPARα results in the proliferation of peroxisomes and the coordinate induction of genes involving the β-oxidation in peroxisomes and mitochondria [33]. Stimulation of fat metabolism by ingesting meal rich in unsaturated fatty acids was confirmed in animal studies. The hamsters fed a meal rich in monounsaturated fatty acid increased mRNA expression of hepatic acyl-CoA oxidase compared to that fed meal rich in saturated fatty acid [34]. Also, the activity of hormone-sensitive lipase in adipocytes of rats ingesting a meal rich in unsaturated fatty acids was higher than that ingesting a meal rich in saturated fatty acids [35]. In an in vitro study, in which various fatty acids were added to cell culture medium for 24-h and conformational changes and binding PPARα to DNA were assessed, suggested that unsaturated fatty acids are more effective in activating PPARα than saturated fatty acids [32]. Additionally, the saturated fatty acid-enriched meal significantly increased insulin secretion compared with the monounsaturated fatty acid-enriched meal [36]. It is plausible that differences in fat oxidation between the two dietary conditions may have been due to differences in insulin levels, although plasma insulin levels were not assessed in the present study. As another potential mechanism affecting fat oxidation, sympathetic nervous system activity was measured in the present study. However, no difference was observed in sympathetic nervous system activity between the two dietary conditions.

Significant difference was observed in parasympathetic nervous system activity between the both meal conditions in the present study. Animal studies reported that sympathetic activity was significantly lower in interscapular brown adipose tissue, pancreas, hypothalamus and cortex in rats fed meal rich in saturated fatty acid than that observed rats fed meal rich in monounsaturated fatty acid [37, 38]. In the present study, sympathetic nervous system activity was not significant difference but parasympathetic nervous system activity was significantly higher in rapeseed oil condition than in palm oil condition. Thus, it is likely that meal rich in monounsaturated fatty acid shifts autonomic nervous system activity toward parasympathetic nervous system activity dominant.

It is worth mentioning the detailed methodology used in indirect calorimetry. Fat oxidation was calculated from VO_2_, VCO_2_ and N according to a chemical reaction equation. In the present study, the adopted equation was based on the oxidation of palmitoyl-oleoyl-stearoyl-triglyceride, the food quotient (FQ) of which was 0.705 [26]. However, fatty acids in the body have a wide variety of chemical structures [39]; therefore, the FQ of triglycerides depends on fatty acid composition. For example, the FQs of tripalmitin, triolein, trilinolein and trilinolenin are 0.703, 0.713, 0.726 and 0.740, respectively. In theory, the chemical reaction equations based on the oxidation of palmitoyl-oleoyl-stearoyl-triglyceride underestimate and overestimate the oxidation of triglycerides composed of unsaturated fatty acids and saturated fatty acids, respectively (see S1 Table). Although the composition of fatty acids oxidised during the calorimetry is unknown, it is reasonable to assume that the ratio of saturated to unsaturated fatty acids oxidised during calorimetry was higher in the meal rich in palm oil compared to that rich in rapeseed oil. In the present study, the difference of accumulated fat oxidation over 24-h between the two dietary conditions was 8.3 g/day, but this difference may have been underestimated due to the technical limitations of the indirect calorimetry. In other words compared to the palm oil condition, fat oxidation in the rapeseed oil condition might have been underestimated, as explained in S1 Table.

This study was the first to demonstrate that a meal rich in rapeseed oil increases 24-h fat oxidation compared with a meal rich in palm oil. Compared to a meal rich in saturated fatty acids, a meal rich in unsaturated fatty acids increases the oxidation of exogenous and/or endogenous fat. The present study findings may have relevance to nutritional interventions aiming to reduce fat mass. However, this study was not without limitations. First, the findings of the present study cannot be extrapolated to provide information regarding the long-term effects of meals rich in saturated or unsaturated fatty acids. When a meal rich in rapeseed oil was consumed, the increase in 24-h fat oxidation was accompanied by decreased carbohydrate condition, although this difference was not statistically significant. If the daily positive carbohydrate balance continues, it would eventually be counterbalanced by autoregulatory increase in carbohydrate oxidation accompanying a decrease in fat oxidation [40]. Alternatively, increased carbohydrate storage under free-living conditions may decrease subsequent energy intake since carbohydrate balance is a strong predictor of subsequent ad libitum food intake [41]. There is one longitudinal study in 43 healthy young adults, in which meal rich in palmitic acid or oleic acid were consumed for 28 days [42]. After 28 days of each meal intervention, body fat was lower in the subject fed rich in oleic acid, compared with that fed rich in palmitic acid. After an evening meal, indirect calorimetry was intermittently performed overnight, and fat oxidation was higher in meal rich in oleic acid than meal rich in palmitic acid. Although 24-h fat oxidation was not estimated, this study suggested a possibility that meal rich in unsaturated fatty acid increased fat oxidation in long-term conditions. Secondly, in order to generalise the present findings on 24-h fat oxidation in male subjects, experiments with female subjects are warranted. In previous study, females responded to a meal rich in monounsaturated fatty acids more than males, via an increase in fat oxidation [43]. Thirdly, when subjects are switched from a low to a high fat meal, it requires a number of days for substrate utilization to equilibrate to the new macronutrient composition [44]. In present study, the experimental meals shifted from 24% (day1) of general fat intake in Japanese to 43% (day2). Therefore, evaluating the effect of dietary meals on fat oxidation and body composition may require a longer intervention period.

In conclusion, the oxidation of exogenous and/or endogenous fat over 24-h was increased by meal rich in unsaturated fatty acids compared with meal rich in saturated fatty acids.

## Supporting information

S1 Table Equation for oxidised lipid and values for oxidation of each triglyceride. (PDF)
