# Supplementary material for: Meal rich in rapeseed oil increases 24-h fat oxidation more than meal rich in palm oil
Source: PLoS One. 2018 Jun 14;13(6):e0198858. doi: 10.1371/journal.pone.0198858 (PMC6002048; doi:10.1371/journal.pone.0198858)
Supplement: S1 Table — (PDF) [file pone.0198858.s001.pdf]

Supporting Information

S1 Table. Equation for oxidised lipid and values for oxidation of each triglyceride.

Triglyceride	Chemical formula	FQ	Caloric equivalent of lipid (kcal/g)	Equation to calculate fat oxidation (g)
Palmitoyl-oleoyl-stearoyl-triglyceride	$C_{55}H_{104}O_6$	0.705	9.50 [26]	(A) 1.67 VO_2–1.67 VCO_2 −1.92 N
Tripalmitin	$C_{51}H_{98}O_6$	0.703	9.36 [45]	(B) 1.68 VO_2–1.68 VCO_2 −1.93 N
Triolein	$C_{57}H_{104}O_6$	0.713	9.47 [46]	(C) 1.72 VO_2–1.72 VCO_2 −1.98 N
Trilinolein	$C_{57}H_{98}O_6$	0.726	9.39 [47]	(D) 1.83 VO_2–1.83 VCO_2 −2.10 N
Trilinolenin	$C_{57}H_{92}O_6$	0.740	9.33 [48]	(E) 1.95 VO_2–1.95 VCO_2 −2.55 N

The theoretical basis of indirect calorimetry, which we followed in the present study, has been described by Ferrannini [26]. This S1 Table clarifies the difference in gross energy by the difference of fatty acids constituting triglycerides. Chemical formula, food quotient, caloric equivalent and equation for oxidation of each triglyceride are shown in S1 Table. Equations for complete oxidation of each triglyceride are different (equation A-E) reflecting differences in its chemical formula. Oxidation of 1 mole of tripalmitin (807.34 g) consumes 1624 L of oxygen and produces 1142.4 L of carbon dioxide. According to equation A and based on 1624 L of oxygen consumption and 1142.4 L of carbon dioxide production, estimated triglyceride oxidation is 804.3 g and it slightly underestimates the

real value (99.6%). Oxidation of 1 mole of triolein (885.432 g) consumes 1792 L of oxygen and produces 1276.8 L of carbon dioxide. According to equation A and based on 1792 L of oxygen consumption and 1276.8 L of carbon dioxide production, estimated triglyceride oxidation is 860.4 g (97.2%). Similarly, oxidation of 1 mole of trilinolein and trilinolenin were underestimated (91.5% and 85.7%, respectively). Thus, equation A, which was used to calculate fat oxidation in the present study, underestimates oxidation of triglyceride composed of unsaturated fatty acids.

Supporting References

45. Linstrom PJ, Mallard, W.G.(eds.) NIST Chemistry WebBook, NIST Standard Reference Database Number 69, National Institute of Standards and Technology. 2017 June 21 [cited 4 June 2018]. In: NIST webbook [Internet]. Gaithersburg MD. Available from: http://webbook.nist.gov/cgi/cbook.cgi?ID=C555442&Units=SI&Mask=2#Thermo-Condensed.
46. Linstrom PJ, Mallard, W.G.(eds.) NIST Chemistry WebBook, NIST Standard Reference Database Number 69, National Institute of Standards and Technology. 2017 June 21 [cited 4 June 2018]. In: NIST webbook [Internet]. Gaithersburg MD. Available from: http://webbook.nist.gov/cgi/cbook.cgi?ID=C122327&Units=SI&Mask=2#Thermo-Condensed.

47. Linstrom PJ, Mallard, W.G.(eds.) NIST Chemistry WebBook, NIST Standard Reference Database Number 69, National Institute of Standards and Technology. 2017 June 21 [cited 4 June 2018]. In: NIST webbook [Internet]. Gaithersburg MD. Available from: http://webbook.nist.gov/cgi/cbook.cgi?ID=C537406&Units=SI&Mask=2#Thermo-Condensed.

48. Linstrom PJ, Mallard, W.G.(eds.) NIST Chemistry WebBook, NIST Standard Reference Database Number 69, National Institute of Standards and Technology. 2017 June 21 [cited 4 June 2018]. In: NIST webbook [Internet]. Gaithersburg MD. Available from: http://webbook.nist.gov/cgi/cbook.cgi?ID=C14465680&Units=SI&Mask=2#Thermo-Condensed.
